# Science or advocacy? The global rise of policy claims and calls to action in population health research (1990-2024)

**DOI:** 10.1093/aje/kwag196

**Published:** 2026-08-14

**Authors:** David Bann, Mengyao Wang, Neil M Davies, Liam Wright, Meredith O’Connor, Emilie Courtin

**Affiliations:** Centre for Longitudinal Studies, University College London, London, United Kingdom; Department of Biostatistics, Yale University, New Haven, CT 06510, United States; LSE Health Inequalities Lab, Department of Health Policy, London School of Economics and Political Science, London, United Kingdom; Division of Psychiatry, University College London, London, United Kingdom; Department of Statistical Science, University College London, London, United Kingdom; Department of Public Health and Nursing, Norwegian University of Science and Technology, Trondheim, Norway; Centre for Longitudinal Studies, University College London, London, United Kingdom; Melbourne Children’s LifeCourse Initiative, Murdoch Children’s Research Institute, Melbourne, Australia; Department of Paediatrics, The University of Melbourne, Melbourne, Australia; Faculty of Education, The University of Melbourne, Melbourne, Australia; LSE Health Inequalities Lab, Department of Health Policy, London School of Economics and Political Science, London, United Kingdom

**Keywords:** bibliometrics, health policy, large language models, metascience, policy making, research impact, researcher advocacy, science-policy interface

## Abstract

Should original research articles routinely contain prominent policy claims or broad calls to action? Growing emphasis on research impact might be welcome yet have unintended consequences (eg, incentivizing overextrapolation and undermining the perceived objectivity of scientists). We examined 45 807 abstracts from 10 leading Epidemiology and Public Health journals (1990-2024). Using a large language model with human validation, we classified policy claims and mapped trends. Claims markedly increased from 17.6% to 35.8%, with wide variation across countries and journals (>60% vs <4%). Keywords linked to higher claim rates differed by topic and time: some corresponded to topics with clear causal evidence of harm as well as topics with notable advocacy. Claims were most common in qualitative or cross-sectional studies, and less common in cohort, quasi-experimental, or experimental studies. We argue that these patterns reflect a research culture increasingly oriented toward claiming policy relevance—and incentives that encourage attaching claims to single studies. Our findings raise questions about how scientists and journals balance evidence, advocacy, and scientific credibility. Ensuring that policy claims and calls to action remain commensurate with evidence will be central to building trust as policy impact continues to be incentivized.

## Introduction

High-quality evidence is required to inform policymaking. This recognition has led to researchers TeX being increasingly expected to have “impact” alongside conducting world-class research. This is coupled with the legitimate desire from researchers to contribute to improving population health, in what has been termed a “consequentialist epidemiology.”^1^ In fact, impact—on policy, society, or the economy—is now a key metric by which universities are awarded funding (eg, in the United Kingdom^2^ and Australia^3^ since 2009-2011). It also informs how researchers are rewarded such as via career progression,^4^ personal awards,^5^,^6^ or grants (eg, National Science Foundation in the United States^7^). This has been termed the “impact agenda,” a term applied to developments evidenced since at least the late 1990s.^2^ There are many competing definitions and frameworks to assess the impact of research on policy.^8^ For example, impact in the United Kingdom is defined as “the demonstrable contribution that excellent research makes to society and the economy.”^9^

However, concerns have arisen about how the “impact agenda” is operationalized (eg, in research policy or funding), and the incentive structures it entails.^2^,^10^ These incentives may have unintended consequences, including operating at cross-purposes with the more traditional aim of academic research: the dispassionate pursuit of truth.^11^ Researchers, who often lack formal training in policy analysis and operate in highly competitive environments, are increasingly rewarded for showing policy relevance. This, in turn, might encourage “bold” or exaggerated impact claims. Indeed, over recent decades, researchers have increasingly used “hyped” (hyperbolic or promotional) language in their research papers,^12^ funding applications,^13^ and impact reports.^14^,^15^

One possible consequence of this trend is the risk of making policy recommendations that extend beyond what a single study, or even a particular study design, can credibly support.^16^ Such overstatements can mislead policymakers and the public about the strength and totality of evidence on a given issue, potentially leading to ineffective policy decisions and a broader erosion of trust in both scientists and public institutions. Beyond these concerns, the “impact agenda” raises additional issues, including potential threats to academic freedom (as research priorities shift toward incentivized short-term policy goals), the transformation of scientists from seekers of knowledge into policy advocates, and the increasing bureaucratic burden of demonstrating impact.^17^

There has been little empirical investigation into the prevalence and evolution of policy claims and calls to action in empirical research papers. A 1999 paper that sampled 3 US-based Public Health/Epidemiology journals found that 24% of papers made policy recommendations.^18^ This differed by journal; *Epidemiology,* a journal with an explicit editorial policy to avoid policy recommendations in research articles, made the fewest claims (8%).^19^ However, this study precedes the rise of the “impact agenda” and does not track changes across time or location (country). It is also limited by the small number of journals, which restricts our understanding of how policy claims have evolved over time and across countries, topics, and research designs.

We thus analyzed policy claims and calls to action in published abstracts from Epidemiology and Public Health journals spanning 1990–2024. Our aim is descriptive: to quantify trends in policy claims (broadly defined) within the impact agenda. We do not assess the validity of individual claims. Our contribution is also methodological: to systematically map policy claims across articles, we employed a frontier open-weight large language model (LLM), which has high performance in a range of relevant benchmarks (eg, scientific understanding),^20^ and may enable efficient classification at low cost.

## Methods

### Journal identification and abstract/metadata retrieval

We included multiple established or newly established Epidemiology and Public Health journals which publish original empirical research. We focus on abstracts of original research articles since (1) they are freely accessible to both policymakers and the public (unlike full texts), and (2) their length makes them suitable for large-scale algorithmic analyses. We expanded upon the 3 journals included in Jackson et al.^18^ (which used manual evaluation) by including non–US focused journals (eg, *European Journal of Epidemiology*/*European Journal of Public Health*/*The Lancet Public Health*). Given the lack of an agreed metric for journal quality or influence, author judgment and discussion informed this choice, resulting in 10 journals (see Table 1).

**Table 1:** Characteristics of Epidemiology/Public Health abstracts: policy claims and calls to action by year, journal, and country.

	**1990-1999**	**2000-2009**	**2010-2019**	**2020-2024**	**All years**
Number of articles, *n*	10 436	12 529	15 464	7378	45 807
Number of unique journals, *n*	9	9	10	10	10
Words in abstracts, mean (SD)	205.5 (67.5)	215.9 (55.2)	221.0 (52.0)	238.4 (57.4)	218.9 (58.5)
Papers with keywords included, %	49.4	60.6	45.1	57.8	52.4
Countries of first author, *n*	112	118	121	104	153
Policy claims, *n* (%)	1841 (17.6)	2857 (22.8)	4394 (28.4)	2643 (35.8)	11 735 (25.6)
Journals, policy claim rate (%)					
Epidemiology	1.2	4.1	4.5	5.3	3.7
European Journal of Epidemiology	19.4	15.3	10.0	14.3	14.4
American Journal of Epidemiology	8.2	8.6	11.1	15.6	9.9
International Journal of Epidemiology	15.3	20.0	16.4	20.0	17.4
Journal of Epidemiology and Community Health	20.8	25.9	30.5	41.1	28.8
European Journal of Public Health	28.1	33.5	40.4	45.2	38.7
Preventive Medicine	25.1	30.3	38.1	45.2	35.7
American Journal of Public Health	24.5	33.7	38.6	46.2	33.7
American Journal of Preventive Medicine	31.3	37.2	35.9	47.2	38.1
The Lancet Public Health	N/A	N/A	59.1	64.1	62.5
Country (first author), policy claim rate (%)					
Norway	8.5	6.3	14.5	16.9	10.8
Japan	9.0	17.3	17.6	18.5	16.0
Denmark	12.8	9.1	11.3	24.9	13.4
Sweden	14.0	15.2	21.9	27.4	18.7
Germany	19.5	17.5	16.5	28.9	19.5
Finland	10.6	18.7	25.8	30.5	20.2
China	11.4	16.8	23.3	33.0	25.8
Netherlands	14.6	22.1	25.1	34.2	22.8
United Kingdom	16.6	23.1	25.5	34.3	24.7
Spain	16.2	24.6	26.8	34.4	25.3
France	17.0	19.9	25.3	34.5	23.5
Canada	16.9	23.1	29.9	34.8	26.9
United States	18.2	24.1	30.5	37.6	26.9
Australia	23.6	26.4	29.7	40.1	29.7
Italy	14.9	19.7	36.0	43.5	23.7

Countries shown are the 15 countries contributing the largest numbers of included articles across the full analytical dataset (1990-2024), ordered by claim rate in 2020-2024; Countries are based on the first author’s country affiliation.

Focusing on articles published from 1990 to 2024 to capture periods before and during the impact agenda, we downloaded all abstracts and relevant metadata (Scopus ID, digital object identifier, title, year, keywords, citation count, journal, article type, country of first author) using the Scopus Application Programming Interface.^21^ Since our goal was to examine original research articles, we extracted only entries listed as “research articles.” We filtered further based on title and keyword fields to remove systematic reviews/meta-analyses or commentaries (*n* = 50 533 records screened; *n* = 4725 records were excluded); one additional record did not contain an LLM policy-claim label (due to an unknown error in the LLM response and was excluded from analyses; analytical sample *n* = 45 807). See Table S1 for details of our sample selection.

This observational study was not preregistered. The primary research question was established a priori (change across time); secondary analyses (eg, country-level differences and topic comparisons) were exploratory.

### Defining policy claims and calls to action using large language models

We employed LLMs to identify policy claims in abstracts. Because LLMs excel at semantic understanding, they may reliably detect policy recommendations at scale. These recommendations are often expressed through varied language patterns and use contextual nuances that can evade simple pattern-matching techniques.

We used the Deepseek v3.1 LLM, a high-performing open-weight model. The use of open-weight models facilitates reproducibility since model weights can be saved, while closed-source models can be deprecated or changed without user control; they also enable analysis in local (non-internet connected) environments. We chose a temperature of 0.1 (temperature is a measure of variability in model outputs; values range from 0 to 1.5 with values closer to 0 ensuring greater determinism in results at the expense of model flexibility). For each abstract, the LLM was prompted to identify explicit or implicit policy recommendations. Prompt engineering principles (eg, specifying the LLM role and giving examples)^22^^,^^23^ were used, which have been found to increase LLM performance; the specific prompt used is shown in Table S2. This was developed iteratively on a small sample of abstracts before being checked for validity with a human classified sample (see below).

A statement within an abstract was defined as a policy claim if it directly suggested policy attention or calls for action, regulation, or intervention. Such statement could either be specific (eg, “States should ban X”) or vague in nature (eg, “This has implications for policy…”). Furthermore, the claim could be directed at a specific group, such as governments or public health organizations, or be more general (eg, “future policies should”). We did not specify to whom the policy claim was directed at; this could thus be policymakers, organizations, or other groups. Finally, to distinguish it from background information, the statement needed to appear in the abstract’s concluding sentences. Statements were explicitly not considered policy claims if they were a suggestion for future research, a finding or statement of fact with no call to action, or a background statement used for motivation.

#### Validation of LLM classification

We validated the LLM classification approach in 4 stages. First, we assessed output stability by repeating classification 3 times on a random sample of 200 abstracts using the same model and parameters (temperature, model version, with random seeds not specified). Run-to-run reliability was almost perfect (Cohen’s *κ* = 0.90-0.98; *n* = 200), indicating high consistency across repeated runs.

Second, we compared LLM classifications with human judgments. Reviewers were given a spreadsheet containing the sampled records (including title, abstract, journal, and keywords) and were asked to record their own judgment for each abstract. Coauthors were not fully blinded, as LLM outputs were accessible in both spreadsheets and the GitHub repository. Inter-rater reliability between the 2 lead human reviewers (author 1 and 5) was high (*κ* = 0.90; *n* = 204). For context, a recent review of concordance in systematic review abstract screening in human raters reported *κ* = 0.82.^24^ Concordance between the LLM and individual human reviewers was also high: author 2 (*κ* = 0.88; *n* = 104), author 1 (*κ* = 0.77; *n* = 204), and author 5 (*κ* = 0.80; *n* = 204). Raw percentage agreement across these human–LLM comparisons ranged from 91.2% to 95.2%.

Third, we created a gold standard set by manually resolving discrepancies across all human-reviewed abstracts. Concordance between the LLM and this adjudicated reference standard remained high (*κ* = 0.80; *n* = 204; sensitivity 76.8%; specificity 98.6%). Results were similar after manually excluding nonempirical papers (*κ* = 0.81; *n* = 197; sensitivity 76.9%; specificity 98.6%).

Finally, to validate findings examining trends in policy claims across time using LLMs to classify abstracts, we (authors 1 and 5) manually reviewed a second sample of 400 abstracts stratified evenly across time (1990-2024), with the order of abstracts randomized and both LLM classification and publication year removed before review (see Results).

### Analytical strategy

#### Policy claims and calls to action by time, country, and journal

We calculated the fraction of abstracts by classification status: overall, and then by year, country, and journal. We first did this in absolute terms (# per year, cumulatively), since the cumulative total of papers that make policy claims may be impactful for evidence translation. We next estimated the claims made in relative (percentage) terms overall and in each subgroup by year, using the analytical sample. To avoid excess variation over time by focusing on single years, we report statistics using categories (1990-1999, 2000-2009, 2010-2019, 2020-2024).

#### Comparison of study designs

To identify whether policy claims differed by study design, we estimated study design using the recorded keywords and title/abstract text. We first searched the title and abstract for prespecified study-design phrases (eg, “cross-sectional study,” “cohort study,” “natural experiment”); if no match was found, we then searched the recorded keywords. Studies were classified as experimental, quasi-experimental, cohort, cross-sectional, ecological/time-series, case-control, or qualitative, and abstracts not matching any prespecified design terms were left unclassified. More details on the search terms and classification details are shown in Table S3. A study design was assigned for 15 075 abstracts (32.9% of all 45 807 abstracts). We compared with a manually reviewed set of 399 abstracts (stratified equally by study design) which showed high concordance (*κ* = 0.84).

#### Comparison of research topics

To assess whether policy claims differed by research topic, we compared keywords listed in abstract metadata by policy classification status; 23 981 had keywords (52.4%). We described the percentage of policy claims among the most commonly reported keywords and among those with the highest rates of policy claims.

To understand the nature of the policy claims made in the abstracts, we randomly selected 100 articles for which we manually checked the content of the claims and classified it as either: (1) broad call for action; (2) further investment in existing policies or intervention; (3) call for new policy direction; (4) focus on specific subgroups/potential new beneficiaries of the policy or intervention; (5) new evidence for policy-making in the area of interest; (6) methods recommendations; and (7) extensions of existing policies.

### Additional and sensitivity analyses

To examine whether differences in claims were driven by changes in publication type, we conducted 2 sensitivity analyses: (1) excluding methodological rather than substantive research (eg, abstracts with keywords such as “epidemiologic methods” or “simulation”), and (2) excluding formal policy evaluations (eg, abstracts with keywords such as “policy analysis”). A decline in the former or an increase in the latter could have upwardly biased any increase in claim rates. We also examined trends using an alternative approach: using regular expressions to classify bold policy claims rather than an LLM. This used phrases in the abstracts (eg, “policymakers should,” ``policymakers must,” “government must,” “stronger policies are needed”); unlike an LLM, this approach is deterministic rather than probabilistic. While our piloting work suggested this led to a vast underestimation of policy claims owing to differing wording across abstracts (kappa in the sample reviewed by humans was 0% since it did not capture any policy claims in the *n* = 100 reviewed by humans), the regular expression approach may still be helpful to triangulate the main finding. Finally, to examine if differences in policy claim rate by study design were robust to the algorithm used to classify study designs, we repeated analyses across multiple approaches (using titles/abstract only, keywords only, combined; and combined but excluding studies with multiple potential study designs).

## Results

### Policy claims and calls to action by time and country

Across the study period (1990-2024), 25.6% of abstracts were classified as making a policy claim, broadly defined. This increased continuously over time: from 17.6% in 1990-1999 to 35.8% in 2020-2024 (Table 1 and Figure 1; Tables S4 and S5). An increasing trend was also found in the 400 abstracts manually classified, with the prevalence of policy claims increasing from 19.3% (95% CI, 13.1-27.5) in 1990-1995 to 56.1% (43.3-68.2) in 2020-2024 (*P*(overall difference) < .001; Table S6 and Figure S1).

**Figure 1:**
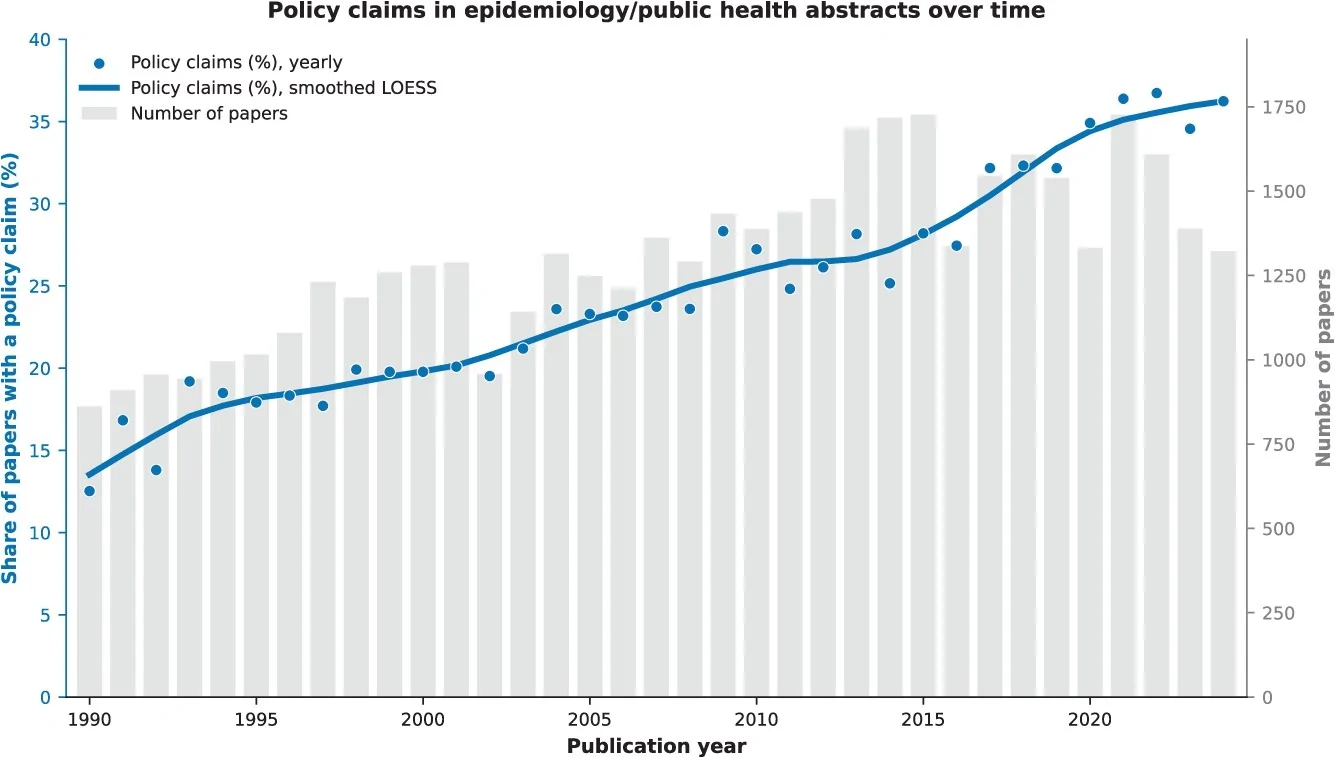
Share of abstracts with policy claims and calls to action in Epidemiology/Public Health Abstracts, 1990-2024.

The prevalence and time trends of policy claims markedly differed by country of first author (Table 1 and Figure 2; Figure S2). For example, among the 15 countries which published the most papers, Italy (43.5%) and Australia (40.1%) had the highest prevalence in 2020-2024, with Japan (18.5%) and Norway (16.9%) the lowest. Italy had the largest percentage point increase from 1990-1999 to 2020-2024 (14.9%–43.5%) and Norway had the lowest (8.5%–16.9%).

**Figure 2:**
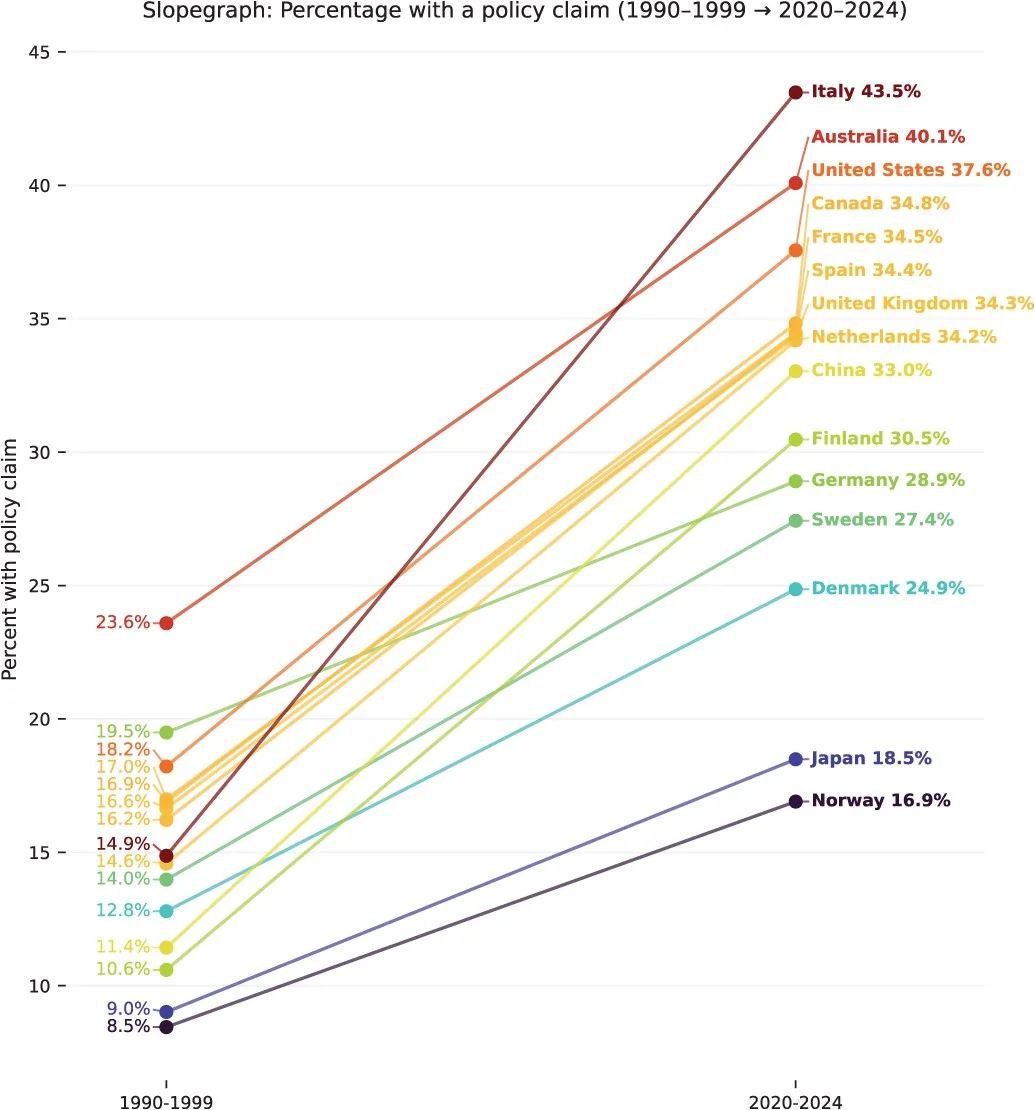
Share of abstracts with policy claims and calls to action by country, 1990-2024.

### Policy claims and calls to action by journal

There was a striking difference in the frequency of claims made by journal: an over 16-fold difference, for example, comparing *Epidemiology* (3.7%) with *The Lancet Public Health* (62.5%); Table 1. We also find clear heterogeneity in the change across time: for example, the *Journal of Epidemiology and Community Health* increased from 20.8% to 41.1%, while claims made in the *European Journal of Epidemiology* declined from 19.4% to 14.3% (Figure 3 and Table 1). In absolute and relative terms, public health–focused journals had higher claim rates than epidemiology-focused journals (Figure 3).

**Figure 3:**
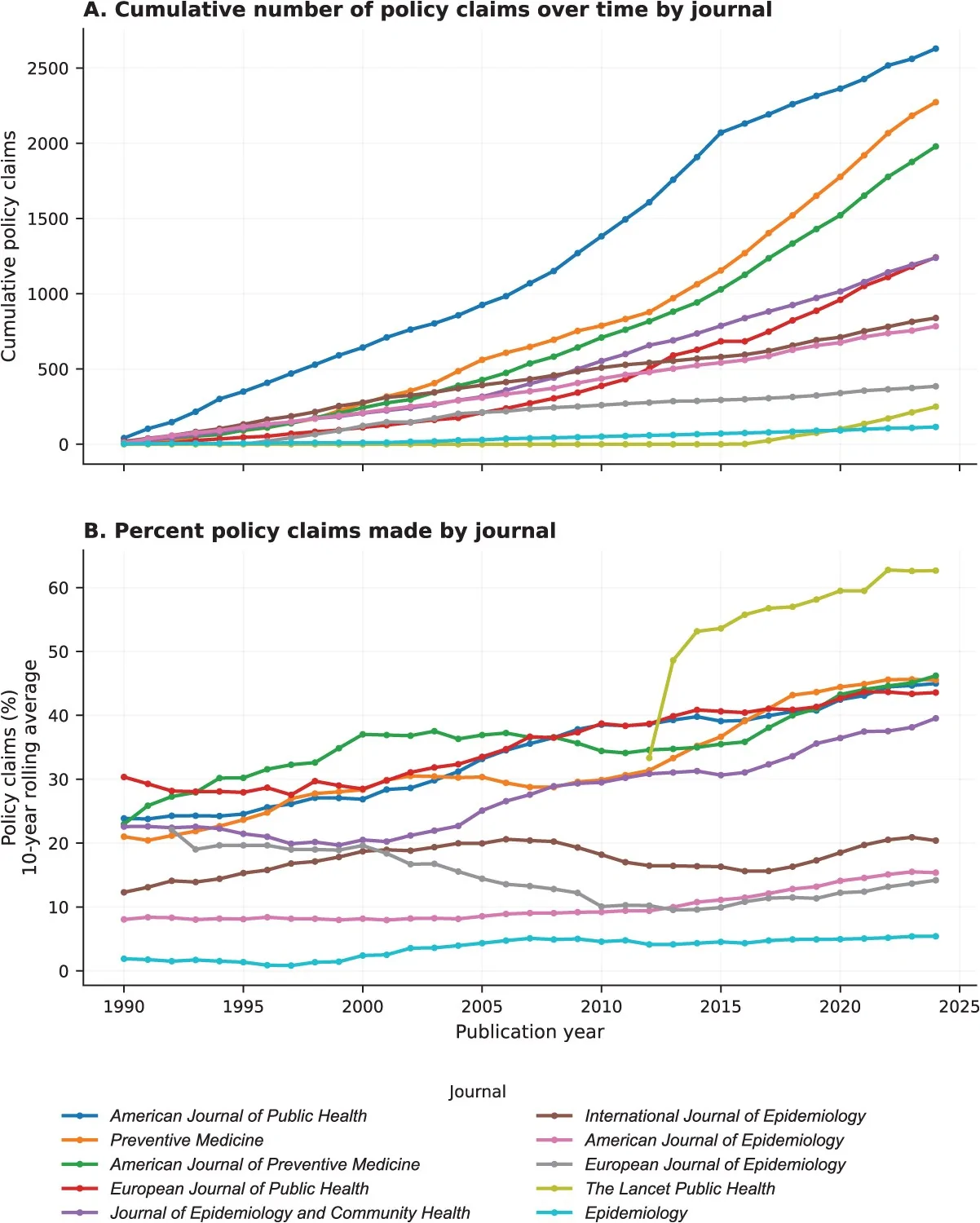
Share of abstracts with policy claims and calls to action by journal: in absolute terms (top) and relative percentage terms (bottom; denominators are the total abstracts published by each journal in each year).

### Policy claims and calls to action by study design

Among the subset of papers which could be algorithmically identified as having a clear study design, policy claims were most frequent in qualitative studies (46.7%), followed by cross-sectional studies (36.9%); Figure 4. Experimental (eg, randomized controlled trials [RCTs]) and case-control studies had the lowest proportion of policy claims. The higher claim rates in qualitative and cross-sectional studies were found across all algorithm specifications (using information in the titles/abstracts or keywords), and in the manually reviewed subset (Figure S3a and b).

**Figure 4:**
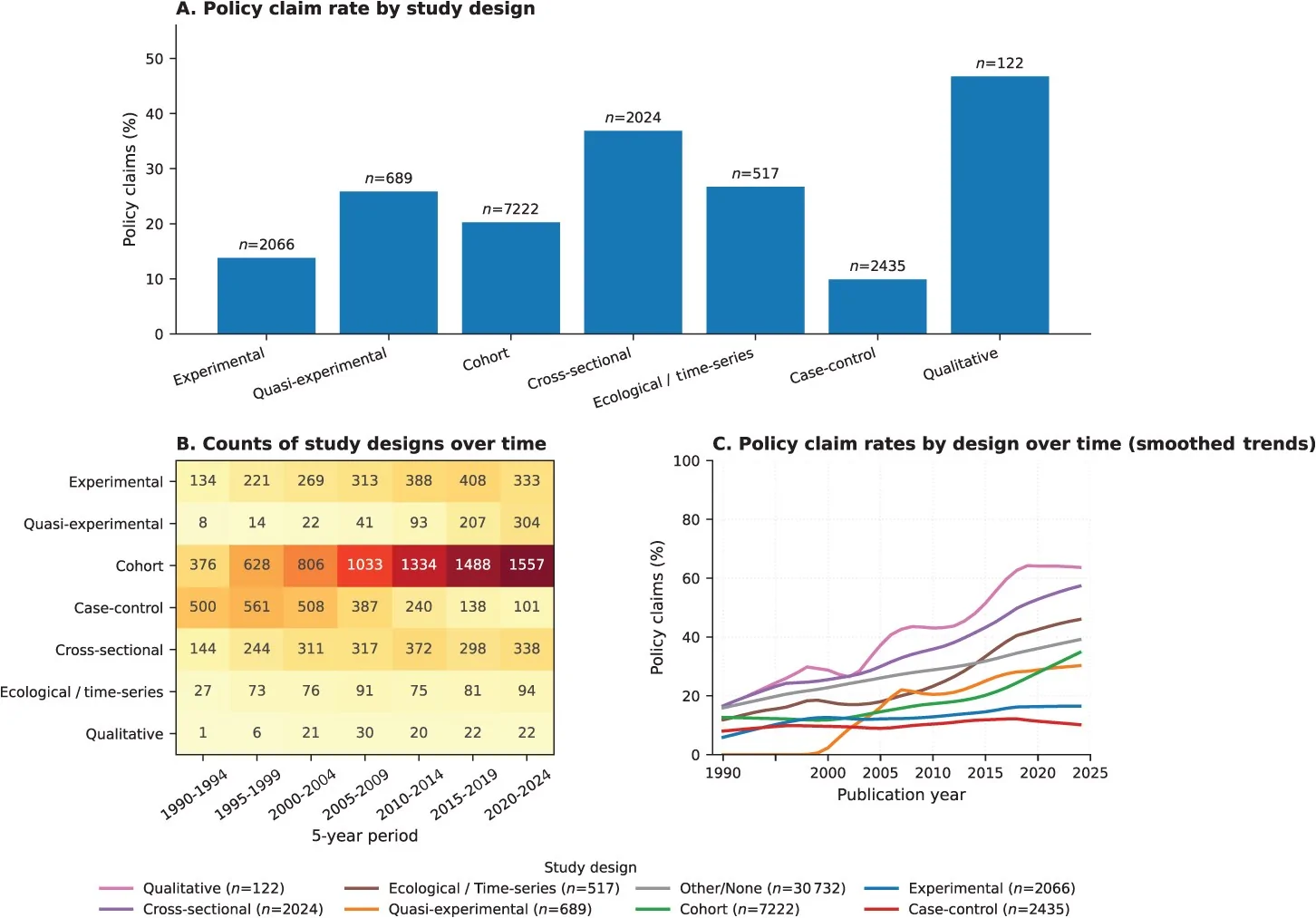
Share of abstracts with policy claims and calls to action, by study design (upper and right panels), and tabulations of study design by year (left). Denominators for % policy claims are abstracts with the estimated study design, across all years (upper panel) or within each year (right panel).

### The characteristics of claims: keywords and types of claims

The rates of policy claims differed by article keywords. Policy claims were, for example, higher than average for fields with clear causal evidence of harm (eg, tobacco, 26.2%) and for topics with notable policy focus and researcher advocacy for example health inequalities (41.3%), poverty (39.8%), and COVID-19 (38.5%) (Figure 4). Keywords with the highest prevalence of policy claims differed across time (eg, research on adolescence and smoking cessation featured prominently in 2000-2009 vs mental health and health inequalities in 2020-2024; Figure S4).

A manual screen of 100 abstracts identified multiple types of claims (Table S7). After excluding 3 claims where it was unclear if a policy claim was made: 38% highlight calls for policy change (eg, wage-setting policies for food insecurity), 27% recommend that policies should differentially target or tailor interventions to sub populations (eg, older migrants), and 25% flag their empirical findings as relevant or important for policy audiences (eg, depression’s link with employment). The remaining include calls for action (5%), calls for further investment in existing policies (1%), and extensions of existing policies (1%).

### Additional and sensitivity analyses

The substantial rise in policy claims was also found when (1) removing methodological (3.7%) and policy evaluation (1.2%) papers (Tables S8 and S9); and (2) when using regular expressions for bold policy claims rather than an LLM to classify policy claims (eg, “policymakers should,” ``policymakers must,” “government must,” “stronger policies are needed”; Figure S5).

## Discussion

We observed a marked increase in policy claims, broadly defined, in published Epidemiology and Public Health abstracts from 1990 to 2024. This coincides with the rise of the impact agenda, with particularly large increases in countries notably affected, such as the United Kingdom, Australia, and the United States.^25^^,^^26^ The sharp rise in policy claims in Italy (19.7% in 2000-2009 to 36.0% in 2010-2019), while not anticipated a priori, is consistent with the 2010 introduction of metrics in Italian academia, which reportedly led to undesired consequences (eg, increasing self-citation rates).^27^

The substantial variation in policy claims suggests that scientific reporting practices are sensitive to external pressures; here, a research culture is increasingly incentivized to demonstrate policy relevance.^2^^,^^10^^,^^17^^,^^26^ Differences across journals might be indicative of editorial policy: for example, *Epidemiology*, which explicitly restricts policy claims to commentaries, had the lowest rate, whereas journals that explicitly encourage policy relevance (eg, *The Lancet Public Health*) had rates up to 16-fold higher.

Whether this large increase is supported (reflecting shifts in the research questions chosen) or not supported (reflecting increasing overextrapolation) cannot be precisely determined using our data alone. Discerning overextrapolation requires deep contextual understanding of each abstract’s specific research question. The claims we identified likely span a spectrum of defensibility. Some are justified within the narrow context of their study findings; others are only defensible when interpreted in a broader sense, for example, when the policy claim refers to an accumulated body of evidence; others are broad statements that motivate further policy interest in a subject. While it is generally advisable that single research studies should not lead to direct policy recommendations,^16^^,^^28^ exceptions exist in fields where the evidence base is fundamentally constrained.

Several lines of reasoning, however, raise questions about a potential increase in overextrapolation or in the use of bold policy claims and calls to action. First, the increase in claims was not accompanied by a marked rise in claims from studies using rigorous causal-inference designs (quasi- and experimental studies; rates were highest for qualitative and cross-sectional studies). This would have indicated that claims were increasingly supported by stronger methodological foundations and approaches explicitly designed to assess policy impacts. The absence of this pattern suggests that incentives to signal policy relevance may have outpaced methodological rigor. Second, policy claims were high in topics where advocacy has been a prominent feature,^29^^,^^30^ where overextrapolation concerns have been raised such as in health inequality^31^ or COVID-19^32^ research. Third, increases in policy claims were also observed using an alternative specification that focused on bold terms (eg, “policymakers should”). Fourth, such increases are anticipated given the competitive landscape researchers and journals operate in: previous studies report increases in promotional language when communicating scientific importance in policy impact reports,^14^^,^^15^ papers,^12^ and grant submissions^13^; in a recent anonymous survey of cohort researchers, 80% reported pressure to overstate their research findings to achieve impact.^33^

The potential for a cumulative body of overstated claims to erode scientific credibility is concerning, even if our estimates are upwardly biased. For example, if only 10% of abstracts classified as making a policy claim are extrapolating beyond the evidence they present, this would still be 1174 papers making such claims (Figure 5).

**Figure 5:**
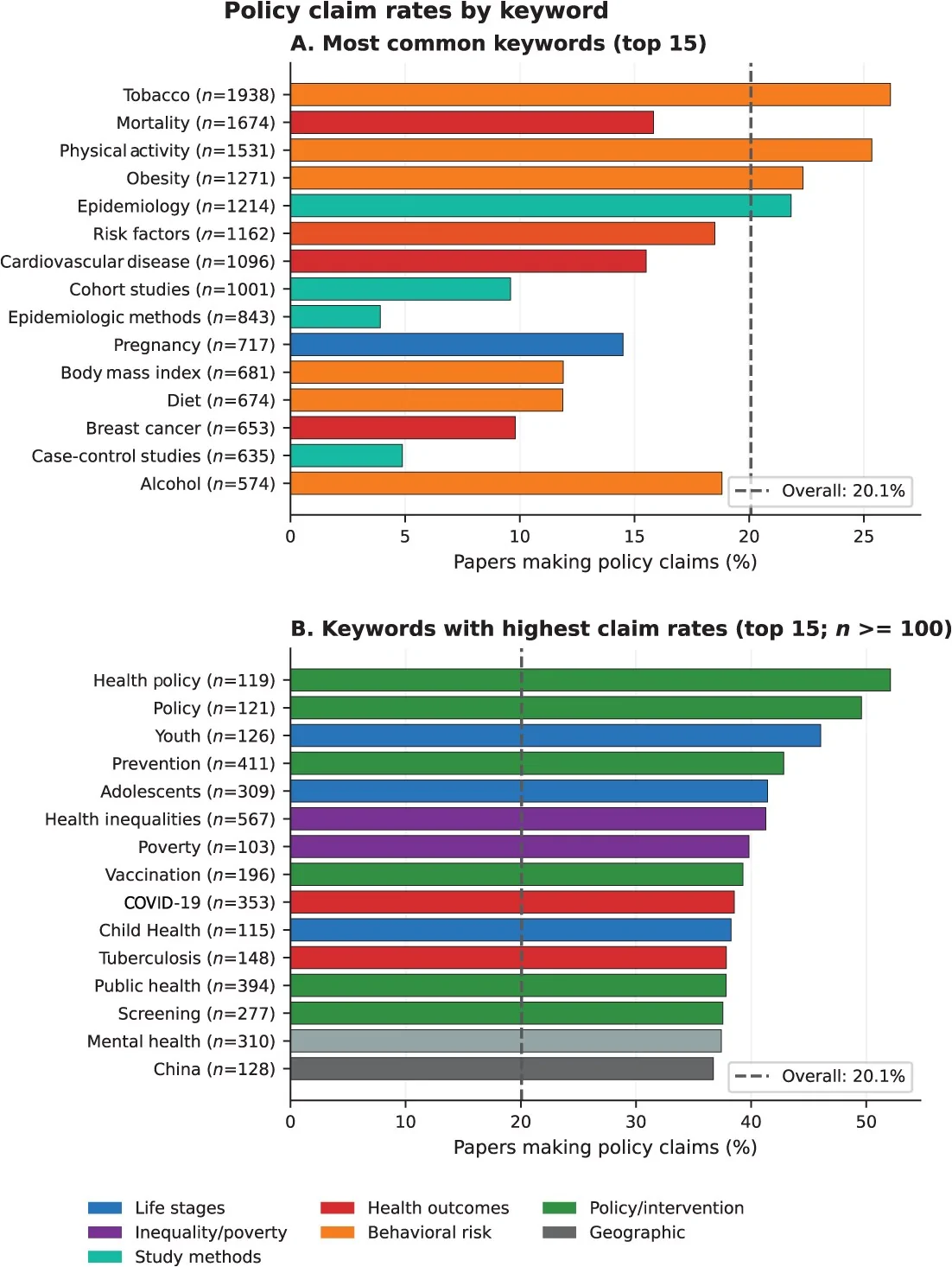
Share of abstracts with policy claims and calls to action by subject (keywords). Denominators are all abstracts with the listed keyword.

A related practical issue concerns where and how policy discussion should be presented to optimally contribute to evidence-based policy; are our publication options optimal to support a robust Epidemiology of consequence? The routine inclusion of policy claims in research abstracts may in part be attributable to the limitations of conventional publishing practices—the legacies of 20th-century paper constraints applied to electronic content. Abstract word limits leave little space for the broader context that authors may implicitly intend (eg, “the collective body of evidence suggests…”). Even in journals that include “evidence summary” or “policy relevance” sections, it is often unclear whether abstract claims refer to the single study or the wider literature. Space within full articles is also limited, restricting opportunities for detailed, evidence-based deliberation of policy options. Alternatives include placing detailed policy discussions in appendices or publishing separate, dedicated policy pieces.^19^

By sharing our methods and code, we aim to encourage further research on the nature, determinants, and consequences of policy communication, and to demonstrate the utility of LLMs for computational metascience. Although copyright restrictions prevent redistribution of the original abstracts obtained from Scopus, we make publicly available metadata along with our LLM classifications. Similar analyses could readily be extended to other disciplines. Economics and other quantitative social sciences, for example, also research population health topics, and economics underwent a “credibility revolution”^34^^,^^35^ marked by a focus on causal identification. By contrast, a recent review of the public health literature found that studies of policy change frequently relied on confounder-adjusted observational approaches rather than quasi-experimental methods.^36^

### Strengths and limitations

Our analysis benefits from the use of LLMs, enabling abstract review at scale and low cost (<$5)—the full population of papers rather than a small sample. This enhances power and representativeness and provides a new resource for future research. Concordance between human and LLM reviewers was high, though both LLMs and human reviewers^37^ make errors. While all such computational approaches may contain errors, such errors would need to vary substantially across subgroups (eg, year and journal) to meaningfully alter our main findings. Triangulation using a regular-expression approach produced qualitatively similar findings (increase in claim rate across time), though it captured substantially fewer policy claims. Additional manual checks also supported the conclusions drawn, for example, a substantial increase in claim rates across time, and higher claim rates in qualitative and cross-sectional studies.

Limitations include the reliance on abstracts, chosen for their accessibility to policy/public audiences and suitability for algorithmic analysis. A comprehensive understanding of impact communication would require examining the full set of researcher outputs, including full texts, press releases, conference presentations, and informal advocacy. Our use of Scopus to extract abstracts reflects both coverage and access constraints; while we piloted the open-access alternative OpenAlex, it lacked full access to copyrighted abstracts. Although we restricted ourselves to articles labeled as “original research,” some nonempirical papers may remain. Reassuringly, sensitivity analyses with additional exclusions (eg, removing abstracts containing methodological keywords) and manual reviews yielded similar findings. Future research could compare policy claims across article types, such as editorials, original research, and reviews, and expand the corpus of included journals (field-specific and general). Finally, identifying study designs algorithmically proved difficult, with only a minority of abstracts being assigned a study design. While the concordance with manual review was high, and the main results were also recovered in those manually classified, future studies could explore improvements in study design classification for example using LLMs. The addition of study design information could add utility to citation databases.

## Conclusion

We observed a marked increase in policy claims, broadly defined, over time, with notable variation across countries, journals, fields of study, and study design. These patterns occurred in the context of a shifting research culture increasingly oriented toward policy relevance—and incentives that encourage attaching claims to single studies. Our findings raise questions about how scientists and journals balance evidence and credibility. We encourage greater discussion of such issues and make our data and code freely available to help catalyze further work.

## Supplementary Material

Web_Material_kwag196

## Data Availability

Analytical code to reproduce the findings, and information on the key datasets used, are available at: https://github.com/dbann/policyclaims.
